# Smart Delayed Fluorescent AIEgens for Organic Light-Emitting Diodes: Mechanism and Adjustable Performance

**DOI:** 10.3390/molecules31020203

**Published:** 2026-01-06

**Authors:** Changhao Yan, Juechen Ni

**Affiliations:** 1 College of Chemistry, Jilin University, Changchun 130012, China; yanch1225@mails.jlu.edu.cn; 2 MOE Key Laboratory of Macromolecular Synthesis and Functionalization, Department of Polymer Science and Engineering, Zhejiang University, Hangzhou 310027, China

**Keywords:** aggregation-induced emission (AIE), organic light-emitting diodes (OLEDs), D-A structures, thermally activated delayed fluorescence (TADF), hybridized local and charge transfer (HLCT)

## Abstract

Organic light-emitting diodes (OLEDs) have attracted remarkable interest in display and lighting. To effectively address triplet exciton harvesting and enhance external quantum efficiency (EQE), delayed fluorescence AIEgens have gained significant prominence. The primary luminescence mechanism involves the efficient harvesting of triplet excitons via reverse intersystem crossing (RISC) channels, categorized into three types: thermally activated delayed fluorescence (TADF), hybridized local and charge transfer (HLCT), and triplet–triplet annihilation (TTA). In this review, we summarize the recent development of doped and non-doped delayed fluorescent AIEgens-based OLEDs. This review mainly discusses the molecular design strategies and photophysical properties of delayed fluorescent AIEgens and the electroluminescent properties of OLEDs as emitting layers. Finally, the challenges and prospects of delayed fluorescent AIEgens for the fabrication of OLEDs are also briefly discussed.

## 1. Introduction

Organic light-emitting diodes (OLEDs) have been widely adopted in display screens of electronic devices (e.g., smartphones, televisions, tablets, and digital cameras) owing to their exceptional advantages, including flexibility, low energy consumption, and high resolution [1,2,3,4]. A typical OLED structure comprises a substrate, anode, hole injection layer (HIL), hole transport layer (HTL), emissive layer (EML), electron transport layer (ETL), electron injection layer (EIL), and cathode [5]. When an external electric field is applied, electrons and holes are injected from the metal cathode and indium tin oxide (ITO) anode, respectively, overcoming potential barriers to migrate toward the luminescent layer. These charge carriers subsequently recombine within the EML to form excitons, which decay radiatively via photon emission [3].

The emissive layer materials can be classified into three types: fluorescent, phosphorescent, or delayed fluorescence materials [6,7,8,9]. Currently, fluorescent and phosphorescent materials dominate OLED applications. However, fluorescent-based EMLs face a fundamental limitation: only 25% of the generated excitons are singlet states capable of direct radiative transition to the ground state, while the remaining 75% are triplet excitons that predominantly undergo non-radiative decay [1,10]. Consequently, the internal quantum efficiency (IQE) of fluorescent OLEDs is theoretically capped at 25%. Phosphorescent materials, though capable of harvesting both singlet and triplet excitons, often suffer from high production costs, further restricting their commercial viability [11].

To address these challenges, extensive research over the past two decades has led to the development of diverse delayed fluorescence (DF) materials, which are broadly classified into P-type and E-type [1,12,13,14]. P-type DF utilizes triplet–triplet annihilation (TTA) to generate singlet excitons, while E-type DF—also known as thermally activated delayed fluorescence (TADF)—achieves singlet-state transitions through RISC [15,16]. Notably, TADF materials offer a theoretical IQE of 100% by harnessing both singlet and triplet excitons, coupled with cost-effectiveness compared to conventional phosphorescent alternatives [17].

**Figure 1:**
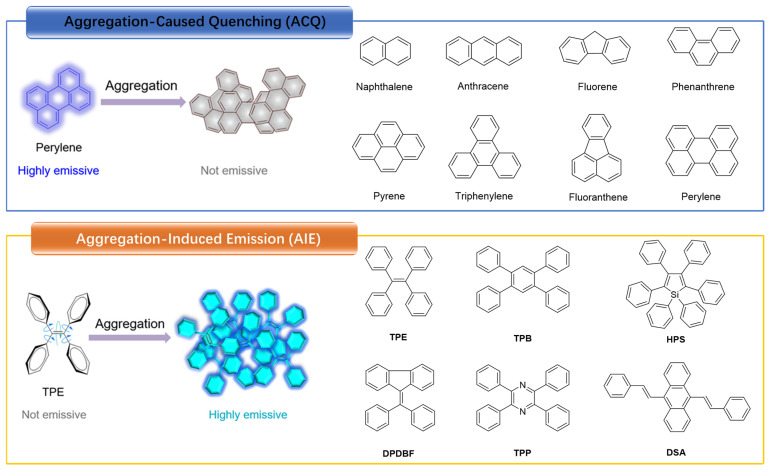
Left: Schematic illustration of ACQ and AIE effect. Right: Molecular structures of typical ACQ fluorophores and AIEgens.

In 2001, Tang’s group proposed the concept of aggregation-induced emission (AIE) [18], and numerous AIEgens have been developed [19,20,21,22,23]. AIE refers to a phenomenon exhibited by luminescent molecules whose structural moieties can rotate or vibrate freely [18]. These molecules typically display weak or no fluorescence in dilute solutions but emit strong fluorescence in concentrated solutions or the solid state (Figure 1). After extensive research, the prevailing mechanism is identified as restriction of intramolecular motion (RIM), encompassing both restriction of intramolecular rotation (RIR) and restriction of intramolecular vibration (RIV) [24,25]. Nowadays, a large number of fluorescent molecules based on tetraphenylethylene (TPE), tetraphenylbenzene (TPB), and tetraphenylpyrazine (TPP) are used in OLEDs [26,27,28]. Given that the light-emitting layer of OLEDs comprises a solid thin film, AIEgens—which demonstrate strong fluorescence in the aggregated state—are more suitable as OLED emitters than conventional aggregation-caused quenching (ACQ) fluorescent molecules derived from the structure unit, such as anthracene, phenanthrene, pyrene, and fluorene [29,30].

Currently, a significant proportion of reported delayed fluorescence AIEgens employed as emissive layer materials in OLEDs is rationally designed based on donor–acceptor (D-A) architectures [31,32]. This design strategy is primarily attributed to the fact that the twisted molecular conformation inherent to D-A structures leads to the spatial separation of frontier molecular orbitals: the highest occupied molecular orbital (HOMO) electron density is predominantly localized on the donor moiety, while the lowest unoccupied molecular orbital (LUMO) electron density is concentrated within the acceptor unit [33]. This orbital separation substantially reduces the overlap between HOMO and LUMO, thereby minimizing the energy gap between the singlet and triplet excited states (*ΔE_ST_*). A small *ΔE_ST_* is a critical prerequisite for efficient RISC, enabling the construction of TADF-type emitters [34]. Furthermore, D-A structures can simultaneously enhance the photoluminescence (PL) efficiency of the locally excited (LE) state component and promote efficient exciton utilization through charge transfer (CT) states, which is pivotal for the development of delayed fluorescence molecules operating via the hybridized local and charge transfer (HLCT) mechanism [35]. From a mechanistic and structural perspective, this review systematically categorizes OLED emissive layer materials into four distinct types: (i) simple D-A structures, (ii) symmetric or asymmetric donor–acceptor-–donor (D-A-D or D-A-D’) architectures, (iii) acceptor–donor–acceptor (A-D-A) systems, and (iv) conjugated frameworks devoid of heteroatom units. Within each category, both doped and undoped delayed fluorescence AIEgens are comprehensively summarized and discussed. The overarching goal of this review is to provide a material-centric foundation and insights for the future development of high-performance, stable luminescent devices. Owing to the scarcity of studies on delayed fluorescence AIE polymers [36,37,38,39], these materials are not encompassed in this review.

## 2. Fundamental Luminescence Mechanism

**Figure 2:**
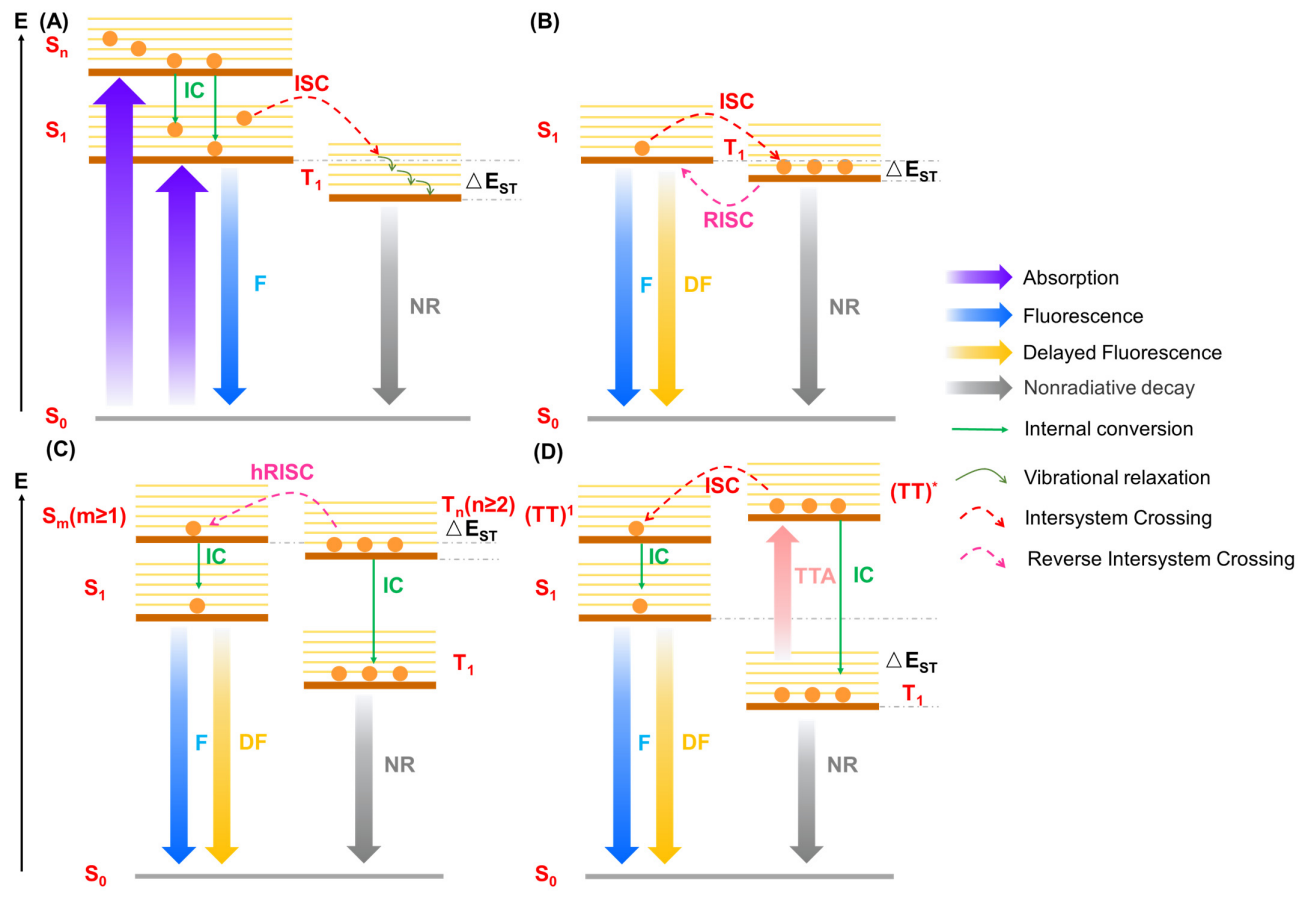
Emission mechanisms in (**A**) fluorescence, (**B**) TADF, (**C**) HLCT, and (**D**) TTA type OLEDs. “*” represents the excited state.

During electroluminescence, light is generated via the recombination of holes and electrons within the light-emitting layer of OLED devices [40,41]. According to spin statistics, the expected ratio of singlet-to-triplet excitons formed by charge recombination is 1:3 [42]. The EQE represents a key parameter for evaluating device efficiency, which is the ratio of photons that escape the device to the injected electrons. It is calculated as *η*_EQE_ = *η*_out_ × *ϕ*_F_ × *η*_r_ × *γ* = *η*_out_ × *η*_IQE_, where *η*_out_ is the optical outcoupling factor (typically approximated as 0.22), *ϕ*_F_ represents the photoluminescence quantum yield (PLQY), *η*_r_ is the efficiency of radiative exciton generation, *γ* is the carrier balance factor, and *η*_IQE_ is the ratio of generated photons in the EML to the injected electrons [43]. The transition of singlet excitons to the ground state produces fluorescence (Figure 2A), and intersystem crossing (ISC) between different electronic multi-states is spin-forbidden, resulting in a theoretical upper limit for the IQE of 25%. To achieve efficient fluorescent materials, the suppression of the non-radiative transitions of singlet excitons is essential. Namely, the fluorescence rate constant (*K_F_*) must be significantly greater than the non-radiative transition rate constant (*K_nr_*). However, even if both *γ* and *ϕ*_F_ approach 100%, the maximum attainable EQE for a fluorescent emissive layer remains limited to approximately 5.5% due to the 25% upper limit on exciton utilization efficiency [44]. Owing to the continuous progress of science, scientists have discovered that surpassing the efficiency limit can be achieved by utilizing triplet excitons directly or converting them into singlet excitons [45,46,47]. The direct radiative decay of triplet excitons manifests as phosphorescence, requiring the incorporation of transition metals such as iridium (Ir), platinum (Pt), and ruthenium (Ru) to significantly enhance the spin–orbit coupling (SOC) effect, thereby boosting phosphorescence intensity [21,37]. However, the widespread commercialization of phosphorescent materials is constrained by the high cost and environmental impact of these precious metals [48,49,50]. Consequently, the effective utilization of triplet excitons through RISC channels presents an alternative strategy. This approach primarily involves three mechanisms: TADF, HLCT, and TTA.

Among them, TADF represents the most promising method for effectively utilizing triplet excitons. This process involves the RISC from the T_1_ to the S_1_ state, assisted by thermal energy when the *ΔE_ST_* is less than 0.1 eV, followed by radiative fluorescence (Figure 2B) [51,52]. Consequently, both singlet and triplet excitons contribute to fluorescence emission, theoretically enabling TADF emitters to achieve an IQE of 100% [53]. However, according to Hund’s rules, the energy of S_1_ is significantly higher than that of T_1_. Thus, triplet excitons in S_1_ undergo facile transition to T_1_ via the ISC process. In conventional fluorescent materials, the rate constant of ISC (*K*_ISC_) typically exceeds that of RISC (*K*_RISC_) by two to three orders of magnitude, rendering the RISC process for triplet excitons kinetically forbidden [54]. Therefore, accelerating the RISC process is key to achieving TADF. A rapid RISC process not only enhances exciton utilization but also reduces triplet exciton concentration, thereby mitigating device efficiency degradation caused by TTA, singlet–triplet annihilation (STA), and triplet–polaron annihilation (TPA) [8]. According to the Boltzmann distribution formula, minimizing *ΔE_ST_* is a crucial pathway to achieving rapid RISC [55]. This can be accomplished by separating frontier molecular orbitals (FMOs). In molecular design, electron donors (D) and acceptors (A) are often introduced simultaneously, where D contributes to HOMO and A to LUMO. Spatial separation of D and A in the molecular structure leads to dissociated HOMO and LUMO distributions, resulting in a smaller *ΔE_ST_* [56,57].

HLCT is a hybrid state, characterized by the coexistence of LE and CT states. Specifically, the low-lying singlet and triplet excited states exhibit LE character with a large *ΔE_ST_*, whereas higher-lying excited states (S_n_ and T_n_, where n ≥ 2) display CT character and minimal *ΔE_ST_*. Analogous to TADF materials, HLCT materials are capable of harvesting triplet excitons, thereby overcoming the intrinsic spin-statistical limit that restricts conventional fluorescent materials to a maximum IQE of 25%. However, a critical distinction lies in their RISC mechanism, as illustrated in Figure 2C. While TADF relies on RISC from T_1_ to S_1_, HLCT materials undergo RISC from higher-lying triplet states to singlet excited states (T_n_ → S_m_, where n ≥ 2 and m ≥ 1) [58]. The IC processes from S_n_ to S_1_ or T_n_ to T_1_ are much faster than other competing processes based on Kasha’s rule [59]. For HLCT materials to efficiently utilize triplet excitons, the high-order RISC (hRISC) process must outcompete the IC pathway. To achieve this, two key energetic criteria must be satisfied, namely a large T_n_-T_1_ energy gap and a small T_n_-S_m_ energy. Additionally, enhancing the spin–orbit coupling (SOC) between highly excited states can also promote the hRISC process, thereby suppressing the IC process and effectively avoiding the annihilation of triplet excitons [60]. Consequently, HLCT-OLEDs can achieve excellent EL performance [7,8,61]. From an experimental perspective, the TADF and HLCT processes can be primarily distinguished by the following two aspects. On the one hand, the HLCT process exhibits a shorter delayed fluorescence lifetime (*τ*_d_), which generally falls in the nanosecond (ns) range. On the other hand, the TADF process suffers from a more significant efficiency roll-off effect than that of HLCT.

For the TTA mechanism, within the theoretical framework, when *ΔE_ST_* (S_1_-T_1_) is sufficiently large, two lower energy triplet excitons can be converted into a higher energy singlet exciton, which returns to the ground state through radiation decay and emits fluorescence (Figure 2D). The fluorescence generated during the TTA process is also known as P-type delayed fluorescence, with a theoretical maximum IQE of 62.5% [62,63]. It is worth noting that the TTA emitters need to meet a huge *ΔE_ST_* between S_1_ and T_1_ and the energy of 2T_1_ should be higher than the energy of S_1_ [64]. Due to strict energy level requirements, molecules with high TTA upconversion efficiency are rare. To differentiate TADF and TTA processes in the experiment, we can compare the following three aspects. First, the most straightforward characteristic is that the TADF process has an ultralow *ΔE_ST_*, which is a parameter that can be precisely quantified using negative ion photoelectron spectroscopy (NIPES), typically less than 0.1 eV. Second, these two processes exhibit distinct correlations between delayed fluorescence intensity and excitation light intensity. The delayed fluorescence intensity of TTA is usually proportional to the square of the excitation light intensity, whereas that of TADF shows a linear relationship with the excitation light intensity. Third, the luminescence intensity of TADF generally rises markedly as temperature increases, while the temperature dependence of TTA luminescence intensity is far less pronounced by comparison.

## 3. AIEgens with D-A Structures

**Scheme 1:**
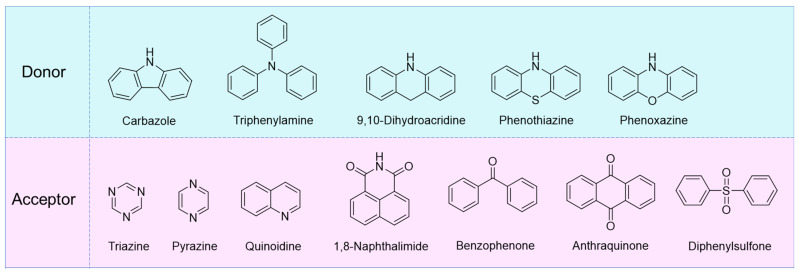
Chemical structures of common donor/acceptor units in D-A type OLED light-emitting layers.

The construction of D-A molecular systems is a proven strategy to enhance the EQE of OLED materials [65,66,67,68]. Donor units typically comprise nitrogen-containing conjugated systems such as carbazole, acridine, and triphenylamine derivatives. Acceptor units commonly incorporate diverse moieties including diphenyl sulfone, diphenylketone, triazine, pyrazine, fused-ring systems, etc. (Scheme 1). Considering the strategic integration of multiple electron-withdrawing structures in electron acceptor design, this review systematically summarizes recent advances from the donor perspective. OLEDs incorporating these structural units primarily operate via the TADF mechanism.

### 3.1. TADF Process

Triphenylamine (TPA), as a classic organic aromatic amine, possesses excellent electron-donating capability and hole transport properties due to the lone pair of electrons on its nitrogen atom. TPA derivatives exhibit high hole mobility and low oxidation potential, effectively facilitating the injection and transport of holes from the anode to the emissive layer. This is crucial for balancing charges within the device, significantly improving luminous efficiency and reducing driving voltage. Meanwhile, the TPA group significantly enhances molecular distortion, thereby preventing fluorescence quenching induced by molecular aggregation. In 2023 [69], Zhang’s group first reported a dual-locked triarylamine derivative donor, which endowed it with numerous unique advantages, such as a lower HOMO level, minimized non-radiative loss, and excellent AIE properties, thereby overcoming the bottleneck in constructing high-performance deep-red/near-infrared TADF OLEDs. Consequently, the resulting emitter DCN-DSP (Entry 1 in Table 1) exhibited enhanced anti-quenching performance, maintaining the optimal OLED performance within a doping concentration range of 5 to 20 weight percent. Its EQE reached 36.2% at 660 nm, 26.1% at 676 nm, and 21.3% at 716 nm. To date, this EQE value is the highest among OLEDs fabricated using triphenylamine as the donor. The photophysical properties of the delayed fluorescent AIEgens with triphenylamine donor units and the performance of the devices using them as EMLs are summarized in Table 1 [70,71,72,73,74,75].

Another group with good hole transport ability, carbazole, can be used as a donor unit and directly connected to the acceptor unit to improve the EQE of the device. Its large steric hindrance is beneficial for suppressing random conformational changes [73,74,75,76,77,78,79,80,81,82,83,84,85,86]. In 2020, Kim et al. synthesized three new color-tunable (from deep-blue to sky-blue) and solution-processable emitters named TB-3Cz, TB-P3Cz, and TB-DACz (Entries 3–5 in Table 2) [76]. These emitters were ingeniously designed by incorporating boron-bridged structures as electron acceptors into different electron-rich hosts, which contain carbazole derivatives as donors. Comprehensive photophysical studies demonstrated that these emitters possess not only TADF properties but also AIE characteristics. Consequently, solution-processable OLEDs fabricated using these new materials as non-doped emitters in the emissive layer exhibited a maximum EQE and Commission Internationale de l’Éclairage (CIE) color coordinates of 9.90% and (0.17, 0.07), respectively, for TB-3Cz; 6.13% and (0.15, 0.08), respectively, for TB-P3Cz; and 6.04% and (0.18, 0.40), respectively, for TB-DACz.

To further improve the performance of OLEDs, Wang et al. developed two D-π-A structured luminescent materials, ICz-DPS and ICz-BP (Entries 6–7, Table 2) [77]. Their doped OLEDs based on ICz-BP provided an excellent electroluminescence EQE (*η*_ext_) and current efficiency (*η*_C_) of 17.7% and 44.8 cd A^−1^, respectively, with an *η*_ext_ roll-off of 2.9%. Their non-doped OLEDs based on ICz-DPS afforded high efficiencies of 11.7% and 30.1 cd A^−1^, with pure-blue emission with CIE coordinates of (0.15, 0.08) and a low roll-off of 6.0%. In 2023, Jiang and co-workers designed and synthesized three novel AIE-active materials with twisted donor–acceptor structures, namely 3phCN-Cz, 3phCN-ICz, and 3phCN-tCzICz (Entries 8–10 in Table 2) [78]. Systematic investigations demonstrated that 3phCN-ICz and 3phCN-tCzICz, which possessed sterically hindered moieties of larger size, exhibited not only excellent thermal and electrochemical stability but also remarkable ability to suppress intermolecular interactions. These characteristics rendered them suitable candidates for constructing high-efficiency solution-processable blue TADF devices. Finally, 4-(tert-butyl)-N,N-dimethylaniline (4TCzBN) was employed as the blue TADF emitter; the solution-processable OLED device based on 3phCN-ICz achieved an EQE_max_ of 22.04%, a CE_max_ of 41.88 cd A^−1^, and a low turn-on voltage of merely 3.5 V. All delayed fluorescent AIEgens with carbazole donor units used as EMLs are shown in Table 2 [79,80,81,82,83,84,85,86].

Compared to triphenylamine, the rotational degree freedom of acridine is greatly reduced, but as a rigid group with large steric hindrance, its introduction into AIEgens can improve material stability [87,88,89,90,91,92,93,94,95,96,97,98,99,100,101,102,103,104,105,106]. In 2022, Yang et al. first successfully developed a series of highly efficient TADF-AIE luminophores based on imidazole–acridine moieties (Entries 1–3 in Table 3) [87]. The introduction of two cyano functional groups onto the imidazole moiety significantly enhanced the electron-withdrawing capability, thereby endowing the TADF emission with a small *ΔE_ST_* value. Meanwhile, the installation of an acridine group at the N1 position of the imidazole moiety twisted the geometric configuration between the imidazole and the benzene bridge, thus converting the imidazole derivative from an ACQ luminophore into an AIE luminophore. Consequently, the non-doped OLEDs employing these TADF-AIE luminophores as emitters exhibited excellent sky-blue and green luminescence, with a high EQE of up to 20.0% and a low efficiency roll-off (EQE measured at 1000 cd·m^−2^ was 16.1%).

Phenothiazine or phenoxazine has similar electronic effects to acridine. However, the photoelectric performance of their derivatives typically surpasses that of acridine derivatives. Table 3 displays TADF AIEgens applied to OLED emissive layers using acridine, phenothiazine, and phenoxazine as electron donors. Two new TADF emitters, BenCN-PXZ and BDCN-PXZ (Entries 4–5, Table 3), which employed an asymmetric donor–acceptor framework, were designed and synthesized by combining different CN-modified acceptor units with phenoxazine PXZ as a strong electron-donating unit in 2021 [88]. The influence of the CN group at different substituting sites was systematically compared to highlight the structure–property relationship. BenCN-PXZ and BDCN-PXZ exhibited red emission with emission peaks beyond 600 nm and short delayed lifetimes within 0.6 µs in neat films. Surprisingly, the delayed lifetime of BDCN-PXZ remained shorter than 1 µs even when doped into the 4, 4′-di(9H-carbazol-9-yl)-1, 1′-biphenyl (CBP) host, implying its promising application in OLEDs. Both non-doped and doped OLEDs were fabricated for comparison. Among them, the doped device based on BDCN-PXZ: CBP demonstrated superior performance and negligible efficiency roll-off (2.8%) at 1000 cd m^−2^, which was nearly the best result reported for orange-red TADF-based OLEDs at that time.

In the same year, Chen’s research team synthesized three quinoline-based TADF molecules, DMAC-QL, PXZ-QL, and PTZ-QL (Entries 6–8 in Table 3), via a one-pot reaction using commercially available materials as the starting substrates. Their twisted conformations were verified by single-crystal X-ray diffraction analysis, and all the compounds exhibited excellent thermal stability. Furthermore, the *ΔE_ST_* values of DMAC-QL, PXZ-QL, and PTZ-QL were determined to be 0.06, 0.10, and 0.04 electron volts, respectively, with corresponding delayed lifetimes of 2.15, 1.86, and 15.76 microseconds, indicating their remarkable TADF performance. These molecules also displayed typical AIE and aggregation-induced delayed fluorescence (AIDF) characteristics in tetrahydrofuran/water (THF/H_2_O) mixed solutions. Non-doped OLEDs based on these emitters were fabricated, achieving maximum EQEs of 7.7%, 17.3%, and 14.8%, along with turn-on voltages of 3.2, 2.6, and 2.8 volts, respectively.

### 3.2. HLCT Process

**Scheme 2:**
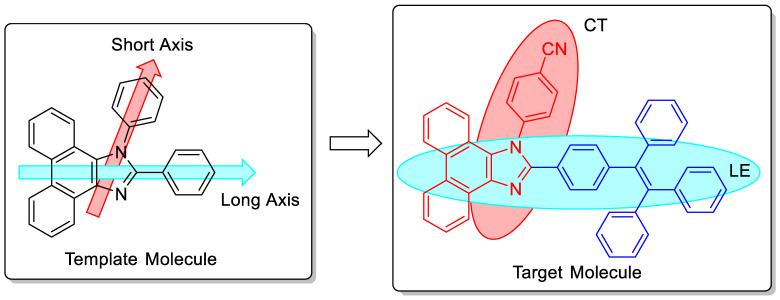
The design method of ppCTPI.

**Figure 3:**
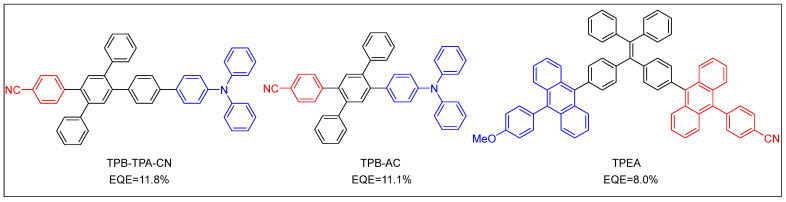
D-A molecular structures of delayed fluorescence AIEgens through TTA process, and EL performance of these AIEgens-based OLEDs.

OLEDs designed using the HLCT process are relatively rare compared to the TADF process. In 2019, through introducing TPE and cyano groups onto an HLCT-type phenanthroimidazole core, Prof. Tang synthesized six luminescent compounds bearing distinct conjugation patterns at the C2 and N1 positions, thereby effectively modulating their excited states [107]. Based on systematic photophysical analysis, the impacts of molecular conjugation patterns on the regulation of LE and CT components were disclosed, and their AIE characteristics that ensure the high PLQYs of these compounds in aggregates were observed. Exciton conversion channels from triplets to singlets via the tuning of excited states were proposed based on theoretical calculations. The non-doped OLED based on 4-(2-(4-(1, 2, 2-triphenylvinyl)phenyl)-1*H*-phenanthro[9, 10-d]imidazol-1-yl)benzonitrile (*pp*CTPI) exhibited excellent performance with a maximum luminance, current efficiency, and EQE of up to 31,070 cd·m^−2^, 18.46 cd·A^−1^, and 7.16%, respectively, and a very small efficiency roll-off of 4.0% at 1000 cd·m^−2^ luminance (Scheme 2). The following year, four naphthothiadiazole-based deep-red emitters with AIE activity were employed as model compounds to reveal referenced structure–property rules in excited states containing large energy level differences between T_2_ and T_1_ levels (*Δ*T_2_T_1_), small *ΔE_ST_* and large SOC, and effective interstate coupling between S_1_ and S_2_ (Figure 3) [108]. These rules were presented as the “Three Golden Principles” because of their acceleration effect in improving emissive exciton transformation from high-level triplet excitons by activating the upper reversed intersystem crossing (uRISC) channel.

In 2021, Yang et al. reported a novel design strategy for constructing intramolecular spatial charge transfer-hybridized local and charge transfer (ISCT-HLCT) materials based on a spiro architecture, where bipolar spirofluorene served as the rigid bridging moiety [109]. Upon doping into a solid-state matrix, 4-phenyl-7-(4-(10-phenyl-10H-spiro[acridine-9,9′-fluorene]-1′-yl)phenyl)benzo[c][1,2,5]thiadiazole (Spiro-2P-BT-TPA) exhibited a high photoluminescence quantum yield of up to 80.9%, which was attributed to its sterically confined D/A stacking structure, multiple intermolecular/intramolecular interactions, and quasi-AIE characteristics. The OLED device based on Spiro-2P-BT-TPA achieved an EQE of 6.6% with a photon utilization efficiency of 40%.

### 3.3. TTA Process

Compared to the delayed fluorescence AIEgens designed for OLEDs using the HLCT mechanism, molecules designed using the TTA principle are rarer. In 2022, Tang’s group demonstrated a novel strategy that employs TTA upconversion materials as triplet quenchers to rapidly deplete the triplet population, thereby suppressing the STA process in AIE-based deep-blue OLEDs [110]. To inhibit the STA process, they doped 1-(2,5-dimethyl-4-(1-pyrenyl)phenyl)pyrene (DMPPP), a material featuring triplet–triplet annihilation upconversion properties, into two AIE emitters, aiming to reduce the triplet excitons at the T_1_ sites of AIE molecules. The results indicated that the resultant blue OLEDs achieved an enhanced EQE of 11.8% with CIE coordinates of (0.15, 0.07), without noticeable efficiency roll-off, thus realizing an efficiency breakthrough for AIE-based deep-blue OLEDs. In the previous year, the same research group successfully fabricated high-efficiency two-color and four-color hybrid white organic light-emitting diodes (WOLEDs) using 4′-(4-(diphenylamino)phenyl)-5′-phenyl-[1,1′:2′,1″-terphenyl]-4-carbonitrile (TPB-AC) as the host material for both blue emitters and yellow fluorophores. Furthermore, by incorporating a triplet–triplet fusion (TTF) layer into the TPB-AC blue emitter, they significantly improved the device efficiency and mitigated efficiency roll-off. Finally, the two-color WOLEDs exhibited a low turn-on voltage of merely 2.6 V, with a maximum EQE of 23.2%, a maximum CE of 73.2 cd·A^−1^, and a maximum PE of 78.7 lm·W^−1^. At a luminance of 1000 cd·m^−2^, the CE, PE, and EQE remained at 63.2 cd·A^−1^, 58.3 lm·W^−1^, and 21.1%, respectively, achieving extremely low efficiency roll-off (Figure 3) [111].

**Figure 4:**
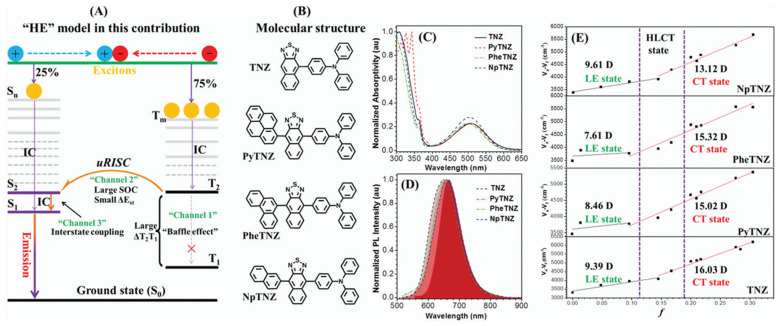
(**A**) Proposed ‘‘HE’’ theoretical model. (**B**) Chemical structures of TNZ-based DR emitters. (**C**) UV-vis absorption spectra of TNZ-based emitters in tetrahydrofuran. (**D**) PL spectra of TNZ-based emitters in tetrahydrofuran (concentration: 10^−5^ M). (**E**) Linear correlation of polarity of solvent media with Stokes shift (*v_a_* − *v_f_*). Adapted and modified with permission from Ref. [107]. (Copyright 2020, Royal Society of Chemistry.)

It is worth mentioning that a deep-blue emitter 1-(10-(4-methoxyphenyl)anthracen-9-yl)-4-(10-(4-cyanophenyl)anthracen-9yl)tetraphenylethene (TPEA) was successfully prepared by a combinational molecular design in 2018, which contains TTF and HLCT characteristics to increase the ratio of triplet excitons used. The TPE moiety contributed to the emitter AIE property (Figure 4), which enhanced the solid-state luminescence efficiency [112]. The crystallographic analysis revealed that the two anthracene groups were twisted relative to the central TPE core, a structural feature that effectively prevented a bathochromic shift in the emission spectrum. In addition, they adopted a donor–acceptor architecture to improve charge balance in OLEDs. The material exhibited high thermal stability, with a glass transition temperature (T_g_) of 155 °C, which was observed during differential scanning calorimetry measurements. Building upon these molecular design advantages, a non-doped OLED device achieved high performance: it demonstrated a turn-on voltage (*V_on_*) of 2.6 V at a luminance of 1 cd·m^−2^, a maximum power efficiency (*η*_PE_, max) of 11.1 lm·W^−1^, and a maximum current efficiency (*η*_CE_, max) of 9.9 cd A^−1^. Notably, the device showed a low current efficiency roll-off even when the luminance reached 1000 cd·m^−2^, indicating excellent operational stability under practical driving conditions. The delayed fluorescence AIEgens with no clear luminescence mechanism in the literature were not introduced in this review [113,114,115,116,117].

## 4. AIEgens with D-A-D (D-A-D’) Structures

The D-A-D structure typically has a more symmetrical molecular configuration, which facilitates the balanced injection and transport of holes and electrons, thereby improving the luminescence efficiency of the device. In addition, symmetrical structures often bring higher glass transition temperatures (T_g_), which helps to improve the thermal stability and film uniformity of materials. This type of device is mainly designed based on TADF and HLCT processes.

### 4.1. TADF Process

Table 4 categorizes donor-dependent AIDF emitters for OLED emissive layers [118,119]. Among them, there are few reports on using triphenylamine as a donor. In 2019, DTPA-DDTM (Entry 1, Table 4) bearing triphenylamine as the donor moiety and phenyl ketone as the acceptor unit was designed and synthesized [118]. Combined charge transfer pathways in a single molecule could be achieved via linking the donor and acceptor units in the ortho-position, which was favorable for reducing *ΔE_ST_* and increasing the luminescence efficiency. The TADF characteristics, AIE properties, solvatochromism, theoretical calculations, and crystallography of the designed emitters were systematically investigated to determine the structure–property relationship. By virtue of the inhibited concentration quenching in the solid state, the non-doped OLEDs employing DTPA-DDTM as the emitter realized high current efficiency LE and EQE of 25.6 cd A1 and 8.2%, respectively.

In recent years, Zhao et al. reported a novel 4,4-difluoro-boradiazaindacene (BODIPY) chromophore featuring a D-A-D structure, designated as 2CF_3_-2TPA (Entry 2 in Table 4) [119]. The introduction of a trifluoromethyl-substituted phenyl group at the meso-position of the BODIPY core enabled the emission shift from red light to far-red light, while the steric hindrance of the trifluoromethyl group suppressed intermolecular interactions. By combining thermally assisted TADF hosts with deep-red dyes, OLED devices were fabricated, which exhibited a far-red turn-on wavelength of 668 nm and an EQE of 2.3%. Given the extensive range of luminescent layer materials containing carbazole, acridine, phenothiazine, or phenoxazine donors, this section focuses specifically on high-performance systems [79,120,121,122,123,124,125,126,127,128,129,130,131,132,133,134,135,136,137,138,139,140,141,142,143,144]. In 2018, a brand-new tailor-made material, namely DCPDAPM (Entry 4 in Table 4) [120], was developed based on a carbazole core, with 9,9-dimethyl-9,10-dihydroacridine serving as the donor moiety and triphenylene as the acceptor moiety. Among the non-doped OLED (Device A) and doped OLEDs (Devices B, C and D), Device D exhibited the optimal electroluminescence performance, achieving a turn-on voltage of 3.6 V, a maximum luminance of 116,100 cd·m^−2^, a maximum CE of 61.83 cd·A^−1^, a maximum PE of 40.45 lm·W^−1^, and an EQE of 19.67%. In 2024, Wang’s research team proposed an efficient molecular design strategy for TADF materials, which involves selecting a highly rigid and planar indolocarbazole donor and a molecularly locked acceptor to construct molecules with a D-A-D configuration, thereby successfully developing luminophores featuring high photoluminescence quantum yield (ΦPL) and small *ΔE_ST_* [121]. These molecules exhibited intense TADF and AIE characteristics in non-doped thin films, accompanied by a ΦPL exceeding 70.0%, small *ΔE_ST_*, low non-radiative transition rate constants, and large K_RISC_. As excellent emitters for OLEDs, 23BCzTPO (Entry 5 in Table 4) enabled highly efficient electroluminescence in the fabricated OLED devices, with CIE coordinates of (0.219, 0.463), a maximum CE_max_ of 36.9 cd·A^−1^, and a maximum EQE_max_ of 21.6%.

For AIEgens with delayed fluorescence using acridine, phenothiazine, and phenoxazine as electron donors, their EQE_max_ applied to OLEDs can reach over 20%. In 2018, Yang’s group prudently designed and synthesized two new derivatives based on quinoxaline as the acceptor and phenoxazine as the donor units, and introduced a fluorine atom owing to its weak electron-withdrawing ability and strong intermolecular electron coupling capability. They anticipated that the two new emitters, namely 10, 10′ -((6-fluoroquinoxaline2, 3-diyl)bis(4, 1-phenylene))-bis(10*H*-phenoxazine) (SFDBQPXZ) and 10, 10′-((6, 7-difluoro-quinoxaline-2, 3-diyl)bis(4, 1-phenylene)) bis(10*H*-phenoxazine) (DFDBQPXZ), can unambiguously embody the AIE-TADF nature and eventually the device efficiency can be remarkably improved with reduced concentration quenching [135]. It is worth mentioning that employing monofluoro-substituted SFDBQPXZ (Entry 20, Table 4) as the emitter in the doped device realized an efficient yellow fluorescent device with the EQE close to 24% without any out-coupling technology and the non-doped device presented an orange emission with the EQE over 10%. In the following years, many AIEgens with D-A-D structures that could achieve EQE_max_ of over 20% were developed and applied to OLEDs [136,137].

**Figure 5:**
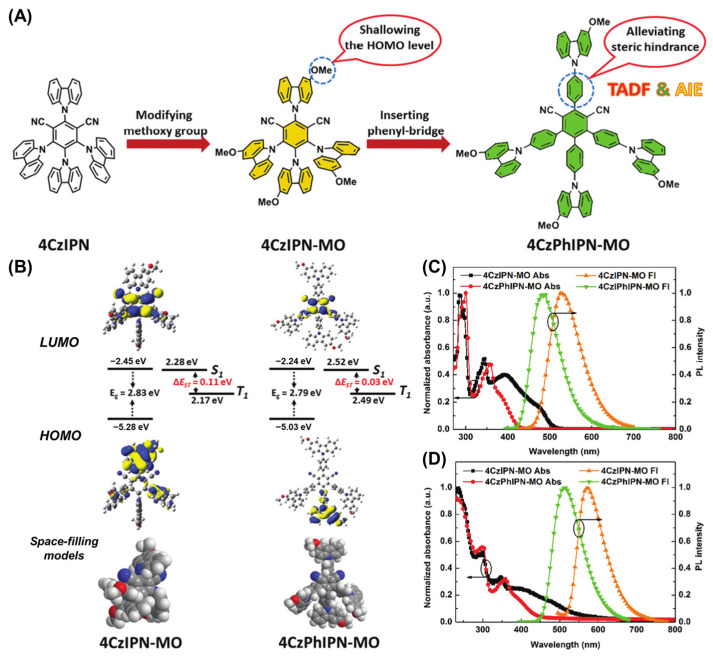
(**A**) Molecular structures of 4CzIPN-MO and 4CzPhIPN-MO. (**B**) Optimized geometries, electron cloud distribution maps, calculated energy levels, and space-filling models for 4CzIPN-MO and 4CzPhIPN-MO. (**C**) UV-vis absorption and PL spectra of 4CzIPN-MO and 4CzPhIPN-MO in toluene solution. (**D**) UV-vis absorption and PL spectra of 4CzIPN-MO and 4CzPhIPN-MO in thin film states. Adapted and modified with permission from Ref. [147]. (Copyright 2020, Royal Society of Chemistry.)

In addition to conventional D-A-D and D-A-D’ AIEgens applied to OLEDs, thermally delayed fluorescence AIEgens with three-arm [145,146,147], four-arm [148,149], and multi-arm [150,151,152] architectures have also been developed based on this design. This type of AIEgens was used for devices with final EQEs ranging from 10% to 20%. For instance, Sun et al. fabricated two tailor-made luminophores, namely 4CzIPN-MO and 4CzPhIPN-MO, both of which exhibited distinct TADF characteristics; in particular, 4CzPhIPN-MO demonstrated remarkable AIE performance (Figure 5) [148]. The benzene bridge structure acted as an axis inserted to mitigate the steric hindrance effect of the donor moieties, thereby endowing the material with AIE properties and suppressing non-radiative decay. Meanwhile, the methoxy groups helped lower the HOMO level, thus reducing the injection barrier in the device. The homogeneous thin films of 4CzPhIPN-MO displayed high solid-state PL efficiency and excellent TADF performance. Consequently, a simple solution-processable non-doped OLED using 4CzPhIPN-MO as the emitter achieved an EQE_max_ of 14.5%, along with substantially reduced driving voltage and efficiency roll-off, and its device efficiency was three times higher than that of the corresponding device employing 4CzIPN-MO.

### 4.2. HLCT Process

All the delayed fluorescence AIEgens designed for OLEDs through the HLCT process are summarized in Table 5 [153,154,155,156,157,158]. Most AIEgens commonly employ benzothiadiazole or naphthothiadiazole as electron acceptors, paired with triphenylamine or TPE as electron donors. As illustrated in Table 5, the EQE of all fabricated devices remains below 10%, notably surpassing the theoretical limit of conventional fluorescent devices yet indicating significant room for improvement. It is worth mentioning that Ma and co-workers designed and synthesized a new D-π-A-π-D type compound of 4,4′-(naphtho [2,3-c][1,2,5]thiadiazole-4,9-diyl)bis(N,N-diphenylaniline) (NZ2TPA), which shows a near-infrared (NIR) emission at 683 nm in neat film state [153]. The non-doped devices based on NZ2TPA exhibited excellent performance, achieving an EQE_max_ of 3.9% with an emission peak at 696 nm and a high luminance of 6330 cd·m^−2^, which were among the highest in the reported non-doped NIR fluorescent OLEDs. Moreover, the device remains a high EQE of 2.8% at a high brightness of 1000 cd·m^−2^, with very low efficiency roll-off. This section lacks a summary of AIEgens with unclear mechanisms [159,160,161,162,163,164].

## 5. AIEgens with A-D-A Structures

**Figure 6:**
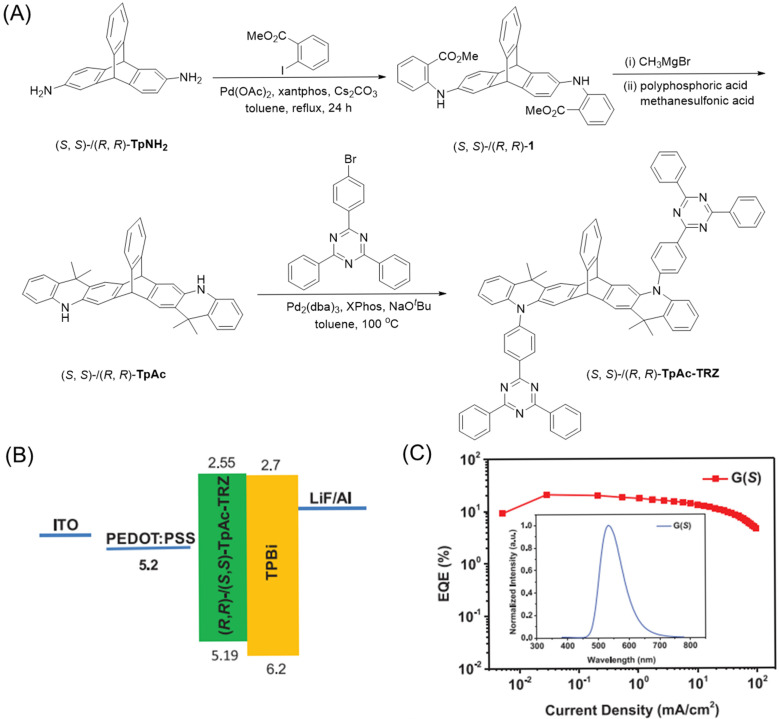

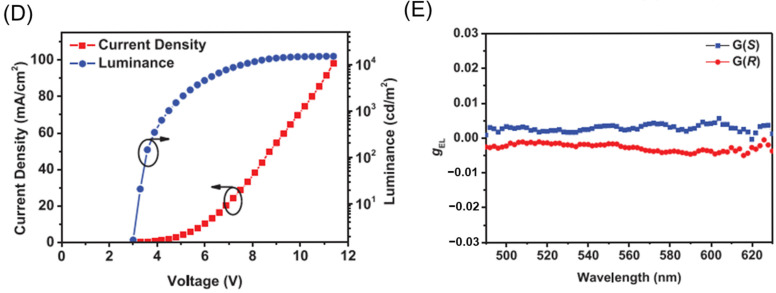
(**A**) Synthetic routes of (S, S)-/(R, R)-TpAc-TRZ enantiomers. (**B**) Energy diagram of the solution-processed non-doped CP-OLEDs based on (S, S)-/(R, R)-TpAc-TRZ. (**C**) EQE–current density characteristic of the device G(S). (**D**) Current density–voltage–luminance (J-V-L) characteristics of the device G(S). (**E**) *g*_EL_ values of CP-OLEDs based on (S, S)-(+)-TpAc-TRZ (G(S)) and (R, R)-(-)-TpAc-TRZ (G(R)) as a function of emission wavelength. Adapted and modified with permission from Ref. [164]. (Copyright 2021, Wiley-VCH.)

The symmetrical A-D-A architecture enables balanced charge transport of both holes and electrons, consequently enhancing the device’s luminescence efficiency. In recent years, there have also been many literature reports on related research [165,166,167,168,169]. Chen et al. reported the first solution-processed non-doped circularly polarized–OLEDs (CP-OLEDs) with high performances based on a pair of triptycene scaffold-based TADF enantiomers (R, R)-(-)-TpAc-TRZ and (S, S)-(+)- TpAc-TRZ (Figure 6A) [165]. The enantiomers have a small *ΔE_ST_* of 0.03 eV and high PLQY of 85%. With the introduction of the triptycene scaffold, TpAc not only acts as a donor unit of the TADF emitters but also as chiral sources featured to induce chirality of the enantiomers. Obvious mirror-image circular dichroism (CD) and circularly polarized luminescence (CPL) signals of the enantiomers were also observed with opposing photoluminescence dissymmetry factors (*g*_PL_) of +1.9 × 10^−3^ and −1.8 × 10^−3^ for (S, S)-(+)-TpAc-TRZ and (R, R)-(-)-TpAc-TRZ, respectively, in neat film. Furthermore, the triptycene scaffold with a rigid 3D structure is conducive to avoiding π-π stacking, thereby inhibiting ACQ in aggregate state. In addition, obvious AIE and AIDF features of (S, S)-(+)-TpAc-TRZ were found, which was favorable for its use as an emitter for non-doped OLEDs. By employing the triptycene scaffold-based TADF enantiomers as emitters, the solution-processed non-doped CP-OLEDs achieved high EQE up to 25.5%, as well as intense CP electroluminescence (CPEL) properties with dissymmetry factors (*g*_EL_) of +1.5 × 10^−3^/−2.0 × 10^−3^ (Figure 6).

**Scheme 3:**
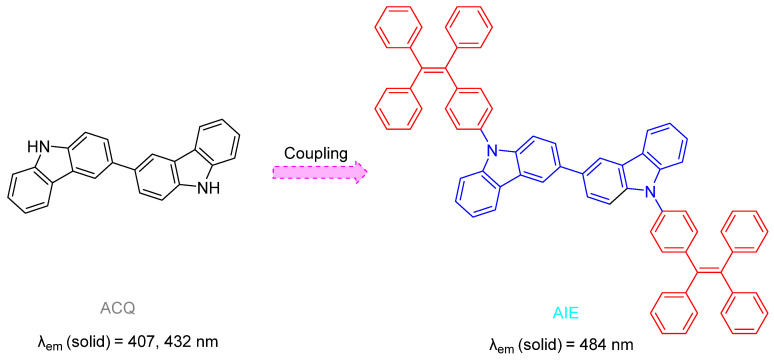
Design strategy to develop bis-carbazole-derived aggregation-induced emissive luminogen.

In 2023, Bose et al. reported the synthesis of bis-carbazole-derived luminogen by oxidative coupling of the carbazole in good yield (Scheme 3) [166]. The bis-carbazole luminogen displayed sky-blue emission in the aggregated state along with excellent electrochemical property and thermal stability, which are considered essential features for constructing high efficiency OLEDs. The luminogen was employed as an emitter for fabricating non-doped OLEDs and the device resulted in sky-blue emission with CIE coordinates (0.23, 0.41). The light-emitting device had a maximum current efficiency of 3.25 cd/A and EQE_max_ of 5%, which eventually rolled-off to about 2% at 50 mA/cm^2^.

## 6. Conjugated AIEgens with Noneheteroatom

**Figure 7:**
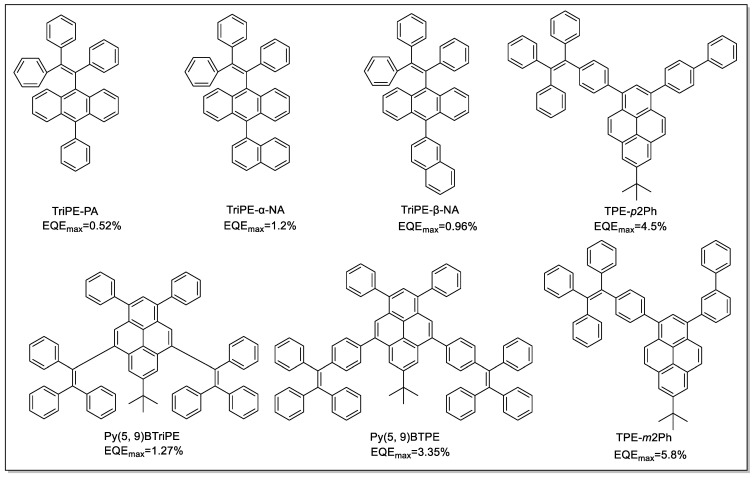
Molecular structures of conjugated AIEgens with noneheteroatom, and EL performance of these AIEgens-based OLEDs.

Although conjugated AIEgens without heteroatoms exhibit relatively low EQE_max_, they remain viable for fabricating stable blue or white OLED devices [170,171,172,173,174]. The molecular structures and EL performance of all conjugated AIEgens without heteroatoms prepared as devices are shown in Figure 7. In 2020, Li et al. synthesized three new TriPE-based sky-blue materials, TriPE-PA, TriPE-α-NA, and TriPE-β-NA, through a simple synthetic route [170]. All new molecules exhibited a typical AIE phenomenon with relatively high αAIE values (25–63) and PLQY (41.7–42.6%) at the aggregation state. These materials were extremely similar in photophysical and electrochemical properties. However, it was found that TriPE-PA performed poorly compared with others, when they were all used as the non-doped emitter in OLED devices. This may be due to the fact that it is a configurationally pure substance, while both TriPE-α-NA and TriPE-β-NA are the mixtures of asymmetric configurations.

In addition, this year, Tang’s group synthesized two pyrene-based hot exciton molecules with the AIE feature by functionalizing the 1- and 3-positions of pyrene, where biphenyl serves as a through-space conjugation (TSC) unit and TPE acts as a rotor [171]. The synergistic effect boosted the blue emission behavior with high PLQY (*Φ*_f_ > 0.39), as well as narrow-band emission in the film state. Using the pyrene-based AIEgens as emitters, non-doped OLED and TADF-sensitizer OLED devices exhibited excellent (pure) blue EL performance. Moreover, the TPA-decorated pyrene exhibited a blue emission (CIE_y_ < 0.13) and narrow full width at half maxima (FWHM) emission (47–60 nm) with high exciton utilization efficiency (>35.6%), while the TPE-decorated system displayed a broader FWHM emission but better OLED performance in both EL devices.

## 7. Conclusions and Outlooks

AIDF materials play an indispensable role in fabricating OLEDs. In the last couple of years, delayed fluorescent AIEgens have attracted great attention due to the high PL efficiency in the aggregated state, which has become the most promising candidate for fabricating OLED devices. This review systematically discusses recent advancements in delayed fluorescence AIEgens for OLEDs, elucidating both three photophysical mechanisms and rational molecular design strategies to accelerate the development of high-performance emissive materials. We describe the construction of AIDF materials from four aspects: D-A structure, D-A-D (D-A-D’) structure, A-D-A structure, and conjugated framework without heteroatoms. To obtain access to the remaining excitons in triplet states, OLEDs based on TADF and HLCT materials have achieved high EQE that is comparable to phosphorescent materials, and the device efficiency of TADF-OLED is generally superior to that of HLCT counterparts. Up to now, the maximum EQE of red AIE TADF-OLED has been increased to 36.2%, whereas it is only 6.8% for HLCT-OLED. However, the HLCT device exhibits relatively low-efficiency roll-off because of the much lower concentration of T_1_ excitons resulting from the fast hRISC process and the inhibited IC of T_n_ − T_1_.

Despite some achievements that have been made in highly efficient delayed fluorescence AIE OLED, the low EQE is still the bottleneck. For TADF materials, the reduced triplet exciton lifetime accompanied with an enhanced RISC rate can effectively reduce the triplet exciton density to alleviate efficiency roll-off. In addition, TADF compounds with a multiresonance effect exhibit advantages of a narrow-emission spectrum, high luminous efficiency, and so forth, which deserves more attention to be able to develop highly efficient luminophore. For HLCT emitters, hole/electron transport materials with matched energy levels should be investigated to further improve EL efficiency.

## Figures and Tables

**Table 1:** Photophysical properties of the delayed fluorescent AIEgens with triphenylamine donor units and the performance of the devices using them as EMLs.

Entry	Emitter Chemical Structures	HOMO ^a^ (eV)	LUMO ^a^ (eV)	*ΔE_ST_* ^a^ (eV)	*λ*_Abs_ ^b^ (nm)	*λ*_PL_ ^c^ (nm)	*τ*_p_ ^c^ (ns)	*τ*_d_ ^c^ (μs)	EQE_max_ (%)	CIE (x, y)	Ref.
1	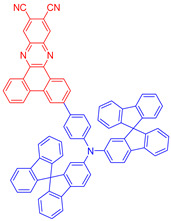	−5.08	−3.00	0.14	-	648	10.7	222	36.2	(0.65, 0.34)	[69]
2	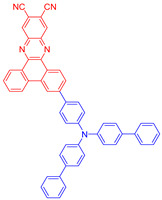	−5.15	−3.01	0.18	-	620	11.2	511	25.2	(0.61, 0.39)	[69]
3	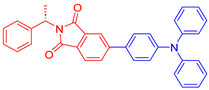	−5.41	−2.33	0.21	299, 396	523	12.4	7.3	2.7	(0.24, 0.52)	[70]
4	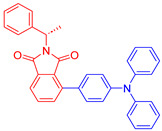	−5.28	−2.31	0.20	307, 339, 392	536	14.8	17.4	10.2	(0.30, 0.54)	[70]
5	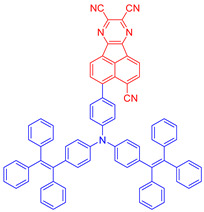	−5.51	−3.94	0.08	605	755	-	16	2.1	-	[71]
6	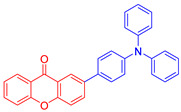	−4.97	−1.75	0.18	395	480	466	154	3.8	(0.18, 0.33)	[72]
7	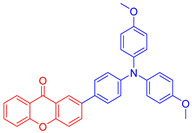	−4.66	−1.67	0.13	410	517	300	77	11.2	(0.30, 0.57)	[72]
8	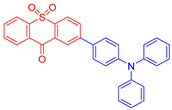	−5.37	−3.49	0.05	450	630	25	78	18.5	(0.45, 0.53)	[73]
9	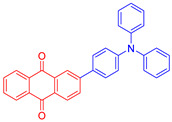	−5.11	−2.69	0.16	288, 327, 462	612	7.56	0.37	7.5	(0.60, 0.40)	[74]
10	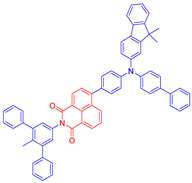	−5.04	−2.29	0.31	343, 447	601	8.3	9.09	7.59	(0.35, 0.60)	[75]
11	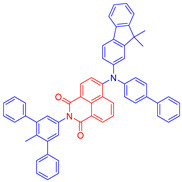	−5.20	−2.30	0.48	332, 484	636	12.4	7.68	7.21	(0.59, 0.41)	[75]

^a^ Obtained from theoretical calculations; ^b^ absorption peaks measured in the different solvents; ^c^ measured in the neat films.

**Table 2:** Photophysical properties of the delayed fluorescent AIEgens with carbazole donor units and the performance of the devices using them as EMLs.

Entry	Emitter Chemical Structures	HOMO ^a^ (eV)	LUMO ^a^ (eV)	*ΔE_ST_* ^a^ (eV)	*λ*_Abs_ ^b^ (nm)	*λ*_PL_ ^c^ (nm)	*τ*_p_ ^c^ (ns)	*τ*_d_ ^c^ (μs)	EQE_max_ (%)	CIE (x, y)	Ref.
1	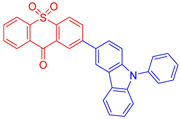	−5.78	−3.58	0.07	420	580	-	-	21.5	(0.31, 0.56)	[73]
2	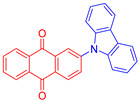	−5.59	−2.89	0.22	284, 336, 439	572	4.53	0.28	5.8	(0.50, 0.49)	[74]
3	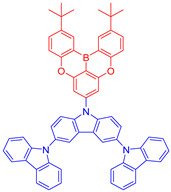	−5.74	−2.68	0.06	294, 378	433	13.7	4.38	9.9	(0.17, 0.07)	[76]
4	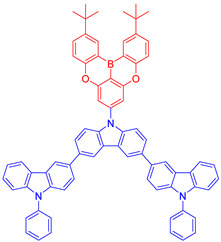	−5.43	−2.57	0.11	305, 380	460	18.5	4.68	6.13	(0.15, 0.08)	[76]
5	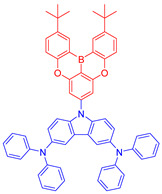	−5.21	−2.48	0.07	306, 379	494	15.7	5.43	6.04	(0.18, 0.40)	[76]
6	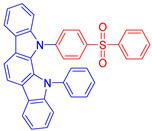	−5.48	−2.38	0.22	290, 324, 362	438	21.6	0.55	11.7	(0.15, 0.09)	[77]
7	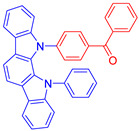	−5.45	−2.45	0.09	281, 292, 325, 365	481	20.3	0.41	17.7	(0.18, 0.30)	[77]
8	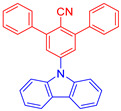	−5.62	−2.19	0.48	339	407	-	-	11.53	(0.15, 0.28)	[78]
9	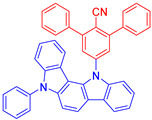	−5.52	−2.20	0.28	362, 345	441	-	-	22.04	(0.16, 0.30)	[78]
10	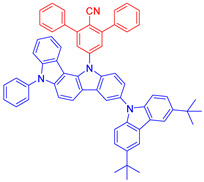	−5.46	−2.14	0.23	362, 331, 298	441	-	-	16.60	(0.15, 0.26)	[78]
11	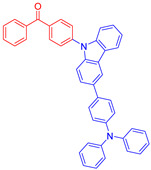	−5.55	−3.16	0.26	335	478	19.7	2.1	3.4	(0.27, 0.43)	[79]
12	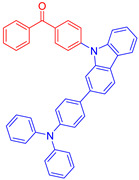	−5.58	−3.15	0.37	350	425, 464	21.2	2.3	1.8	(0.21, 0.29)	[79]
13	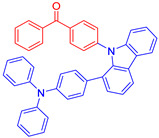	−5.51	−3.18	0.02	345	411, 510	14.2	1.7	7.6	(0.26, 0.42)	[79]
14	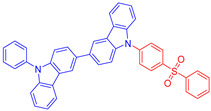	−5.43	−1.96	0.32	302, 343	447	11.2	-	3.66	(0.15, 0.22)	[80]
15	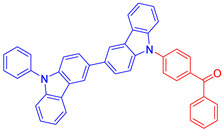	−5.38	−2.21	0.22	304, 352	489	33.1	-	4.69	(0.19, 0.37)	[80]
16	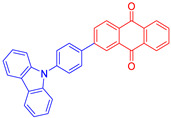	−5.64	−3.19	0.26	-	-	37.6	3.11	5.47	-	[81]
17	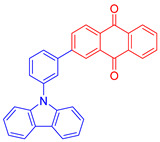	−5.69	−2.96	0.18	-	-	49.1	1.37	3.93	-	[81]
18	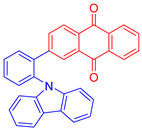	−5.72	−3.24	0.03	-	-	46.3	1.09	22.14	-	[81]
19	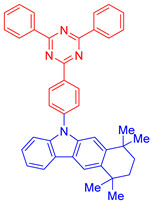	−5.91	−2.85	0.18	288, 298, 347, 379	446	7.3	5.2	7.1	(0.14, 0.14)	[82]
20	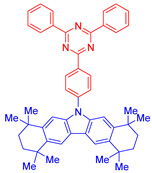	−5.85	−2.88	0.15	287, 297, 347, 378	453	6.6	4.7	14.5	(0.14, 0.15)	[82]
21	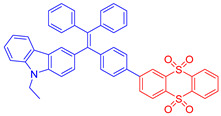	−5.24	−2.08	0.12	284, 303	558	25.4	2.12	-	-	[83]
22	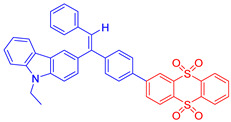	−5.27	−1.88	0.08	285, 300	553	40.9	3.81	-	-	[83]
23	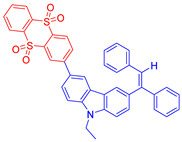	−5.35	−2.09	0.04	320, 357	525	6.24	6.31	-	(0.19, 0.38)	[84]
24	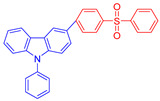	−5.73	−2.51	0.44	301, 325	416	1.82	-	3.2	(0.16, 0.03)	[85]
25	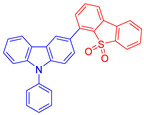	−5.72	−2.56	0.18	291, 345	438	40.6	0.24	7.1	(0.15, 0.10)	[85]
26	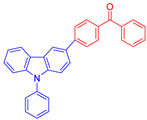	−5.66	−2.57	0.42	-	447	2.11	-	3.7	(0.15, 0.15)	[85]
27	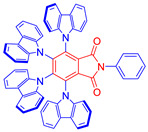	−5.53	−2.67	0.10	373	578	33	1.22	16.7	(0.45, 0.43)	[86]

^a^ Obtained from theoretical calculations; ^b^ absorption peaks measured in the different solvents; ^c^ measured in the neat films.

**Table 3:** Photophysical properties of the delayed fluorescent AIEgens with acridine and similar (phenothiazine or phenoxazine) donor units and the performance of the devices using them as EMLs.

Entry	Emitter Chemical Structures	HOMO ^a^ (eV)	LUMO ^a^ (eV)	*ΔE_ST_*^a^ (eV)	*λ*_Abs_ ^b^ (nm)	*λ*_PL_ ^c^ (nm)	*τ*_p_ ^c^ (ns)	*τ*_d_ ^c^ (μs)	EQE_max_ (%)	CIE (x, y)	Ref.
1	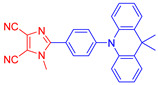	−5.04	−1.87	0.03	354	479	16.7	5.8	15.3	(0.18, 0.33)	[87]
2	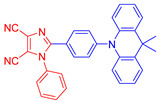	−5.09	−1.95	0.01	366	500	21.3	14.1	13.7	(0.25, 0.50)	[87]
3	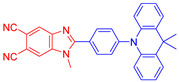	−5.19	−2.21	0.01	362	502	22.8	4.5	20.0	(0.30, 0.53)	[87]
4	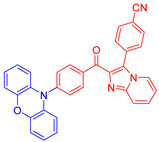	−4.98	−2.61	0.01	330	580	26.3	1.64	13.31	(0.45, 0.53)	[88]
5	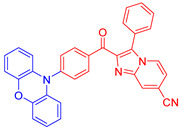	−5.01	−2.69	0.08	330, 440	589	24.0	0.74	13.75	(0.44, 0.53)	[88]
6	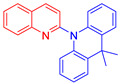	−5.38	−2.21	0.06	350	489	46	2.15	5.9	(0.31, 0.51)	[89]
7	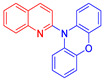	−5.16	−2.16	0.10	365	531	53	1.86	16.1	(0.36, 0.55)	[89]
8	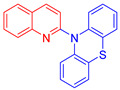	−5.20	−2.01	0.04	350	537	42	15.8	12.7	(0.39, 0.56)	[89]
9	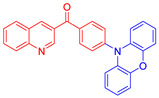	−5.18	−2.71	0.04	421	530	27.4	5.7	11.8	-	[90]
10	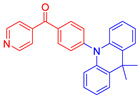	−5.28	−2.64	0.05	290, 370	541	34	1.25	8.4	(0.39, 0.54)	[91]
11	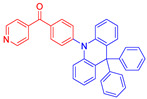	−5.43	−2.65	0.07	290, 370	523	31	1.71	9.7	(0.32, 0.55)	[91]
12	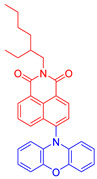	-	-	0.04	344	686	14.7	3.87	4.5	(0.56, 0.36)	[92]
13	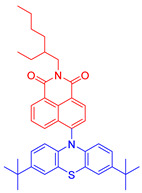	-	-	0.05	347	751	14.9	4.23	2.5	(0.48, 0.49)	[92]
14	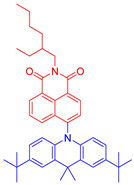	-	-	0.03	348	650	15.1	3.57	8.2	(0.59, 0.39)	[92]
15	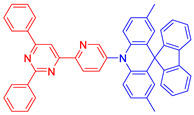	−4.83	−1.98	0.05	397	491	22.6	4.92	19.9	-	[93]
16	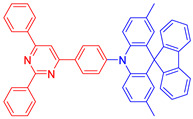	−4.81	−1.82	0.03	419	515	31.5	2.65	18.8	-	[93]
17	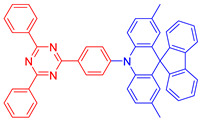	−4.78	−1.98	0.03	407	521	27.5	5.39	21.7	-	[93]
18	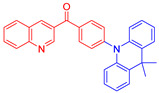	−5.03	−2.21	0.01	355	520	37.8	8.21	11.7	(0.29, 0.57)	[94]
19	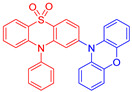	−5.20	−2.42	0.02	304, 324, 380	512	16.1	2.49	16.4	(0.27, 0.50)	[95]
20	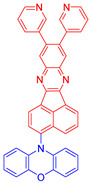	−5.06	−2.68	0.01	329	608	13.6	9.0	15.0	-	[96]
21	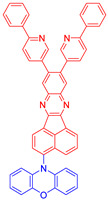	−5.04	−2.66	0.01	340	610	12.4	8.5	19.6	-	[96]
22	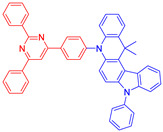	−5.15	−2.53	0.15	315, 390	538	67	0.73	22.0	(0.33, 0.58)	[97]
23	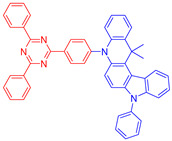	−5.15	−2.68	0.12	315, 400	556	73	0.88	13.2	(0.40, 0.56)	[97]
24	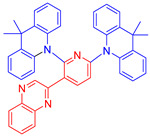	−5.10	−2.42	0.06	285, 372	550	24.1	3.18	11.1	-	[98]
25	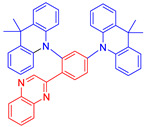	−5.12	−2.44	0.07	285, 364	535	18.6	4.47	9.3	-	[98]
26	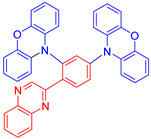	−4.94	−2.44	0.05	320, 400	585	7.81	0.68	4.5	-	[98]
27	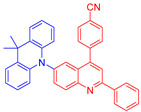	−5.25	−2.44	0.08	401	509	28	39.2	10.3	-	[99]
28	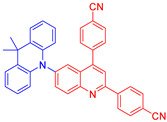	−5.39	−2.80	0.02	422	600	22	1.4	14.6	-	[99]
29	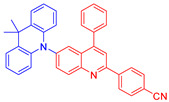	−5.26	−2.51	0.07	411	520	34	49.3	11.9	-	[99]
30	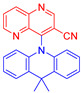	−5.17	−2.67	0.01	320, 498	632	26.8	0.77	5.4	(0.47, 0.49)	[100]
31	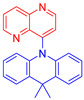	−5.02	−2.02	0.03	309, 433	551	40.0	2.98	8.7	(0.43, 0.54)	[100]
32	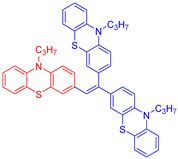	−4.72	−1.22	-	241, 268, 390	539	2.38	-	5.70	-	[101]
33	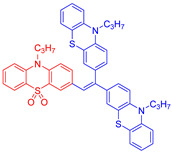	−4.91	−1.42	-	236, 266, 337	534	1.94	-	4.69	-	[101]
34	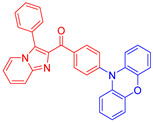	−4.56	−1.96	0.03	325	544	21.4	0.8	5.05	(0.31, 0.57)	[102]
35	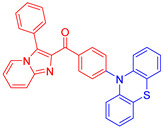	−4.88	−1.94	0.02	345	552	22.3	0.7	15.77	(0.42, 0.57)	[102]
36	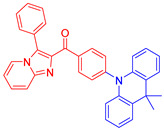	−4.78	−1.95	0.01	355	508	24.8	1.7	11.7	(0.28, 0.57)	[102]
37	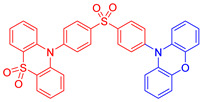	−5.10	−2.41	0.03	315, 400	525	19.1	3.2	11.7	-	[103]
38	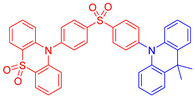	−4.95	−2.04	0.10	320, 375	479	22.9	3.2	11.7	-	[103]
39	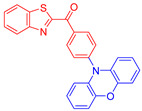	−5.15	−2.91	0.01	317, 458	683	9.15	0.63	1.1	(0.58, 0.41)	[104]
40	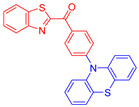	−5.14	−2.33	0.01	304, 389	654	21.8	1.3	1.8	(0.50, 0.46)	[104]
41	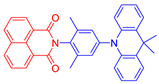	−4.99	−2.91	0.13	322, 330, 350	595	59.9	-	5.38	(0.37, 0.42)	[105]
42	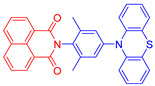	−5.04	−2.94	0.05	325, 350	624	93.0	-	7.13	(0.59, 0.40)	[105]
43	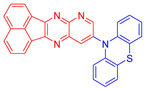	−5.79	−3.68	0.02	322, 418, 485	596	30	3	4.6	-	[106]
44	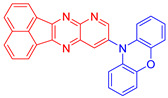	−5.79	−3.68	0.02	322, 472, 566	605	33	14	3.5	-	[106]
45	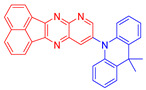	−5.95	−3.62	0.10	322, 447	576	22	116	15.3	-	[106]
46	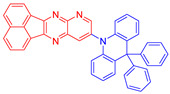	−6.00	−3.64	0.04	322, 440	569	10	200	8.7	-	[106]

^a^ Obtained from theoretical calculations; ^b^ absorption peaks measured in the different solvents; ^c^ measured in the neat films.

**Table 4:** Photophysical properties of the delayed fluorescent AIEgens with D-A-D (D-A-D’) structures and the performance of the devices using them as EMLs (a typical example of structurally similar molecules).

Entry	Emitter Chemical Structures	HOMO ^a^ (eV)	LUMO ^a^ (eV)	*ΔE_ST_* ^a^ (eV)	*λ*_Abs_ ^b^ (nm)	*λ*_PL_ ^c^ (nm)	*τ*_p_ ^c^ (ns)	*τ*_d_ ^c^ (μs)	EQE_max_ (%)	CIE (x, y)	Ref.
	Delayed fluorescent AIEgens with triphenylamine donor units										
1	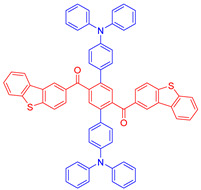	−5.35	−2.74	0.17	301, 404	539	17.8	2.4	8.2	(0.41, 0.55)	[118]
2	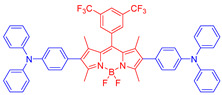	−5.28	−3.34	0.61	309, 455, 561	679	66	0.82	2.3	(0.61, 0.39)	[119]
	Delayed fluorescent AIEgens with carbazole donor units										
3	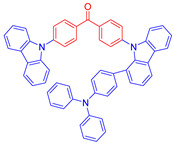	−5.36	3.06	0.04	346	411, 521	13.1	1.6	13.3	(0.29, 0.48)	[79]
4	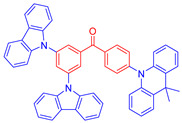	−5.03	−2.21	0.10	288, 336	518	34.2	8.1	19.67	(0.26, 0.56)	[120]
5	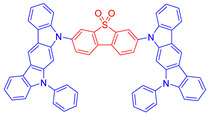	−5.27	−2.39	0.20	305, 346, 363	496	14.6	28.8	21.6	(0.219, 0.463)	[121]
6	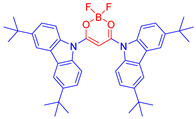	−6.07	−3.19	0.16	380	470	2.10	9.12	18.6	(0.22, 0.42)	[122]
7	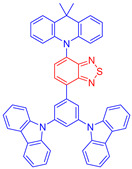	−5.04	−2.78	0.16	290, 337	634	4.94	120	5.6	(0.53, 0.37)	[123]
8	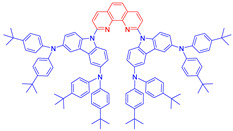	−4.53	−1.59	0.13	365	536	9.9	39	19.7	-	[124]
9	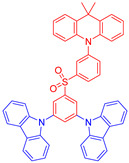	−5.07	−1.83	0.21	-	508	-	1.23	10.05	(0.11, 0.12)	[125]
10	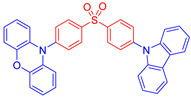	−5.0	−2.4	0.02	300, 340	540	-	1.76	17.03	(0.47, 0.41)	[126]
11	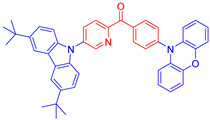	−5.23	−3.04	0.03	387	578	10.1	0.98	6.9	(0.53, 0.46)	[127]
12	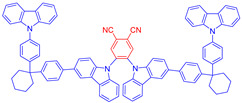	−5.74	−3.06	0.17	287, 330, 399	522	25.2	6.92	13.4	-	[128]
13	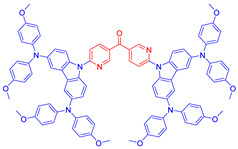	−4.45	−2.06	0.06	449	645	9.1	9.4	0.17	(0.62, 0.37)	[129]
14	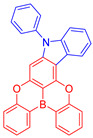	−5.56	−2.59	0.08	300, 316, 346, 382	433	4.29	2.56	9.0	(0.16, 0.18)	[130]
15	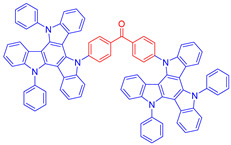	−5.33	−2.77	129	320	520	40.8	0.91	6.0	(0.38, 0.55)	[131]
16	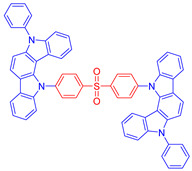	−5.43	−2.28	0.15	306, 318, 343, 361	445	11.5	17.9	13.1	(0.16, 0.14)	[132]
17	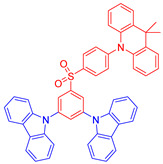	−5.13	−1.85	0.07	285, 334	496	-	0.73	14.27	(0.12, 0.13)	[133]
18	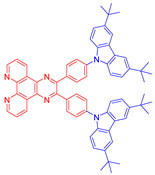	−5.69	−2.88	0.46	299, 387	464	4.3	-	1.7	(0.15, 0.23)	[134]
	Delayed fluorescent AIEgens with acridine, phenothiazine, and phenoxazine donor units										
19	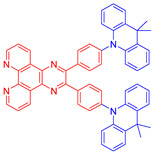	−5.41	−2.83	0.32	303, 363	525	9.3	0.5	5.8	(0.28, 0.52)	[134]
20	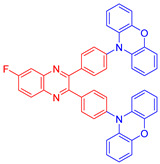	−5.12	−2.52	0.02	414	555	21.6	4.6	23.5	-	[135]
21	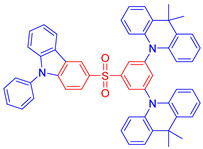	−4.98	−1.47	0.13	288, 336, 365	484	36.4	5.3	20.3	(0.14, 0.15)	[136]
22	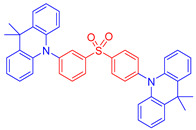	−5.42	−1.88	0.03	279, 370	476	-	5.4	20.5	(0.17, 0.29)	[137]
23	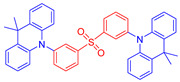	−5.76	−2.79	0.01	280	288	57.9	4.33	16.6	(0.18, 0.30)	[138]
24	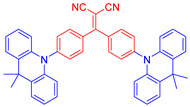	−5.28	−3.22	0.01	505, 590	640	25.1	0.89	12.7	-	[139]
25	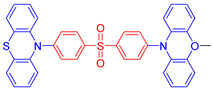	−5.78	−3.06	0.09	209, 241, 314, 379	418	6.2	-	17.7	-	[140]
26	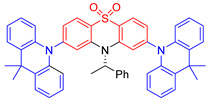	−5.07	−1.48	0.05	340	450	23	3.4	18.5	-	[141]
27	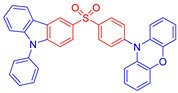	−4.77	−1.41	0.03	290, 312, 380	518	4.0	0.96	14.5	-	[142]
28	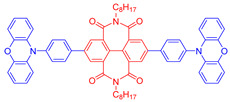	−4.86	−2.89	0.03	310, 387	540	-	20.5	18.1	(0.38, 0.57)	[143]
29	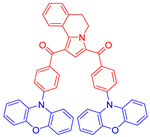	−5.07	−2.85	0.07	332, 418	535	18.4	2.3	16.6	(0.41, 0.55)	[144]

^a^ Obtained from theoretical calculations; ^b^ absorption peaks measured in the different solvents; ^c^ measured in the neat films.

**Table 5:** Photophysical properties of the delayed fluorescent AIEgens through the HLCT process and the performance of the devices using them as EMLs.

Entry	Emitter Chemical Structures	HOMO ^a^ (eV)	LUMO ^a^ (eV)	*ΔE_ST_* ^a^ (eV)	*λ*_Abs_ ^b^ (nm)	*λ*_PL_ ^c^ (nm)	*τ*_p_ ^c^ (ns)	EQE_max_ (%)	CIE (x, y)	Ref.
1	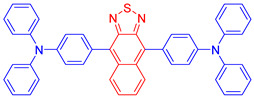	−5.05	−2.95	1.06	582	683	-	2.8	(0.70, 0.31)	[153]
2	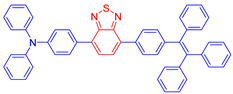	−5.31	−3.23	0.59	314, 446	592	5.12	5.00	(0.53, 0.47)	[154]
3	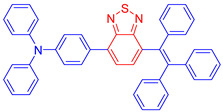	−5.31	−3.12	0.70	309, 432	586	5.61	3.82	(0.49, 0.49)	[154]
4	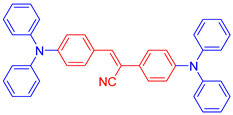	−5.5	−2.5	0.08	305, 430	510	-	6.8	-	[155]
5	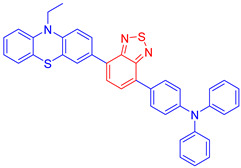	−4.98	−3.10	0.84	314, 461	677	-	1.62	(0.65, 0.32)	[156]
6	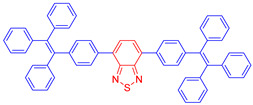	−5.68	−3.13	0.09	414	546	5	4.84	(0.38, 0.56)	[157]
7	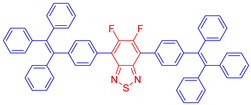	−5.74	−3.25	0.05	393	525	3	4.93	(0.31, 0.53)	[157]
8	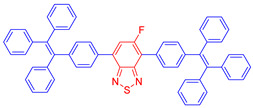	−5.72	−3.16	0.14	401	539	4	4.82	(0.37, 0.55)	[157]
9	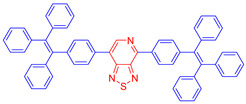	−5.62	−3.41	0.08	451	561	6	4.46	(0.46, 0.54)	[157]
10	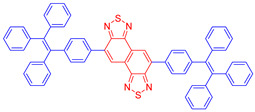	−5.70	−3.30	0.11	441	597	7	5.11	(0.53, 0.46)	[157]
11	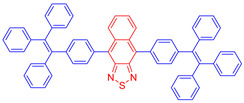	−5.73	−3.51	0.20	489	620	7	3.51	(0.60, 0.39)	[157]
12	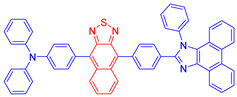	−5.24	−3.22	1.16	311, 506	668	10.38	6.56	(0.64, 0.35)	[158]
13	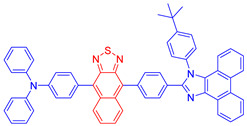	−5.41	−3.40	1.16	311, 506	665	10.2	6.83	(0.67, 0.33)	[158]
14	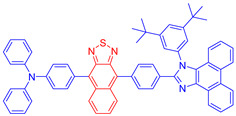	−5.41	−3.36	1.16	311, 506	667	10.37	5.18	(0.67, 0.32)	[158]

^a^ Obtained from theoretical calculations; ^b^ absorption peaks measured in the different solvents; ^c^ measured in the neat films.

## Data Availability

No new data were created or analyzed in this study.
